# The Role of Presepsin and Procalcitonin in Early Diagnosis of Bacterial Infections in Cirrhotic Patients with Acute-on-Chronic Liver Failure

**DOI:** 10.3390/jcm11185410

**Published:** 2022-09-15

**Authors:** Razvan Igna, Irina Gîrleanu, Camelia Cojocariu, Laura Huiban, Cristina Muzîca, Ana-Maria Sîngeap, Cătălin Sfarti, Stefan Chiriac, Oana Cristina Petrea, Sebastian Zenovia, Robert Nastasa, Tudor Cuciureanu, Remus Stafie, Ermina Stratina, Adrian Rotaru, Carol Stanciu, Mihaela Blaj, Anca Trifan

**Affiliations:** 1 Department of Gastroenterology, Grigore T. Popa University of Medicine and Pharmacy, 700111 Iasi, Romania; 2 Intensive Care Unit, “St. Spiridon” University Hospital, 700115 Iasi, Romania; 3 Institute of Gastroenterology and Hepatology, “St. Spiridon” University Hospital, 700115 Iasi, Romania

**Keywords:** presepsin, procalcitonin, bacterial infections, acute-on-chronic liver failure, biomarkers

## Abstract

Background and Objectives: Bacterial infections represent one of the most frequent precipitating events of acute-on-chronic liver failure (ACLF) in a patient with liver cirrhosis (LC). Early diagnosis and treatment could influence the ACLF reversal rate and decrease the mortality rate in these patients. The study aimed to evaluate the role of presepsin, C-reactive protein (CRP), and procalcitonin (PCT) in the early diagnosis of bacterial infections in patients with LC and ACLF, defined according to the European Association for the Study of the Liver-Chronic Liver Failure Consortium (EASL-CLIF) criteria. Material and Methods: We performed a prospective observational study including all consecutive cirrhotic patients with ACLF admitted to our tertiary university center. The patients were follow-up until discharge. All patients were screened for infection at admission, and we included patients with community-acquired or healthcare-associated bacterial infections. Results: In this study, we included 153 patients with a median age of 60 years, of whom 65.4% were male. Infections were diagnosed in 71 patients (46.4%). The presepsin, CRP, and PCT levels were higher in patients with infections than in those without infections (*p* < 0.001, *p* = 0.023, and *p* < 0.001, respectively). The ROC analysis results demonstrated that the best cut-offs values for infections diagnosis were for presepsin 2300 pg/mL (sensitivity of 81.7%, specificity of 92.7%, AUROC 0.959, *p* < 0.001), CRP 5.3 mg/dL (sensitivity of 54.9%, specificity of 69.6%, AUROC 0.648, *p* = 0.023), and PCT 0.9 ng/mL (sensitivity of 80.3%, specificity of 86.6%, AUROC 0.909, *p* < 0.001). Presepsin (OR 3.65, 95%CI 1.394–9.588, *p* = 0.008), PCT (OR 9.79, 95%CI 6.168–25.736, *p* < 0.001), and MELD score (OR 7.37, 95%CI 1.416–18.430, *p* = 0.018) were associated with bacterial infections in patients with ACLF. Conclusion: Presepsin level ≥2300 pg/mL and PCT level ≥0.9 ng/mL may be adequate non-invasive tools for the early diagnosis of infections in cirrhotics with ACLF.

## 1. Introduction

Patients with liver cirrhosis (LC) can develop acute-on-chronic liver failure (ACLF) secondary to an acute liver injury such as acute alcoholic hepatitis, viral infections flairs, or bacterial infections. ACLF is a very dynamic entity, and early recognition and treatment of the precipitating factors could improve patients’ outcomes [1].

Bacterial infections represent the most frequent trigger for ACLF in patients with LC from Europe and North America [2]. ACLF is characterized by an important inflammatory syndrome caused by immune system activation by different self or non-self molecules [3,4]. This systemic inflammation determines multiple organ failure and an increased mortality rate [3]. Moreover, one hour delay in antibiotic treatment in patients with LC and sepsis is increasing the mortality rate by 7.6% [4,5]. Therefore, an early diagnosis and prompt treatment of infections in these patients could increase the survival rate and the chance of a liver transplant. Unfortunately, infection diagnosis is difficult in patients with LC and ACLF because the symptoms are very similar to those of acute decompensated LC, and the patients already have leucopenia, arterial hypotension, hypoxemia due to portal hypertension, and an altered mental status due to hepatic encephalopathy (HE) [6,7,8].

During the last decades, serum biomarkers were developed for the early diagnosis of sepsis in the general population. Presepsin, procalcitonin (PCT), and C-reactive Protein (CRP) are the most used in clinical practice for sepsis diagnosis, although, in LC, the cut-off values are different compared to the general population [3,9,10,11,12].

Presepsin is one of the biological markers that proved to be efficient in the early diagnosis of infections in LC. Presepsin is produced by the cleavage of soluble CD14. CD14 is a co-receptor for bacterial ligands, including the lipopolysaccharide (LPS) complex of Gram-negative bacteria (GNB), making presepsin a biomarker of innate immune activation [13,14,15]. The ability of presepsin to diagnose bacterial infections relies on the fact that after bacterial cellular phagocytosis, presepsin is released into the plasma in direct correlation with the severity of the infection [16].

CRP is a nonspecific biomarker of inflammation that also increases during bacterial infections, being an acute-phase response protein. It is synthesized by the liver as a response to inflammation or tissue damage; therefore, it might be down-regulated in advanced liver cirrhosis or ACLF [12,17].

Procalcitonin has low molecular weight and renal clearance, being influenced by acute or chronic kidney injury. It is synthesized by thyroidian C cells and many other parenchymal tissues as a response to bacterial toxins or inflammatory cytokines, and it was demonstrated as a sensitive biomarker of bacterial infections and sepsis in the general population and cirrhotic patients [18,19,20].

Until now, only two Asian studies evaluated the role of presepsin as a marker of bacterial infections in patients with ACLF, using the Asian Pacific Association for the Study of Liver (APASL) criteria [21,22]. It has to be mentioned that these criteria were developed in a cirrhotic population in whom hepatitis B virus (HBV) flares were the most frequent cause of ACLF, and these results could not be generalized to the European population. Considering all these data, our study evaluated the role of presepsin, procalcitonin, and CRP in the early diagnosis of bacterial infections in cirrhotic patients admitted with ACLF, defined according to the European Association for the Study of the Liver-Chronic Liver Failure Consortium (EASL-CLIF) criteria.

## 2. Materials and Methods

### 2.1. Patients

We conducted a prospective observational study in which we included all consecutive adult patients with liver cirrhosis and ACLF admitted to our tertiary university hospital from 1 January 2020 to 31 December 2020. The patients were follow-up during hospitalization.

The following patients were excluded: individuals without ACLF, patients receiving recent (less than one week) antibiotic treatment, excepting rifaximin, prior admission, patients with severe pulmonary or cardiac comorbidities, those with end-stage malignancies, including hepatocellular carcinoma, individuals with fungal infections, and those with nosocomial infections.

The study received our Local Ethics Committee approval (No. 102/10.10.2019). The study was conducted following the principles of the Declaration of Helsinki. All patients or their legal representatives (for patients admitted in a coma) signed the informed consent before entering the study.

### 2.2. Clinical and Laboratory Assessment

Liver cirrhosis was diagnosed according to the clinical, laboratory, and imaging data. All patients were in a decompensated stage of the disease. The severity of LC was assessed using the Child–Pugh class and Model for End-Stage Liver Disease (MELD) score. ACLF was diagnosed according to EASL-CLIF criteria [2].

The bacterial infections were diagnosed based on clinical manifestations, physical examination, and biological and imaging tests. All patients were screened for infection at admission.

Spontaneous bacterial peritonitis was diagnosed if the absolute polymorphonuclear leukocyte count in the ascites was >250 cells/mm^3^ or if the ascitic fluid cultures were positive. Urinary tract infection would be considered if the urine culture had >10^5^/mL bacterial colony counts. Bloodstream infections would be diagnosed if the patient had positive blood culture. *Clostridioides difficile* infection (CDI) was diagnosed by the presence of stool A and/or B *Clostridioides difficile* toxins in patients with watery diarrhea. Pneumonia was diagnosed based on symptoms and suggestive chest X-ray images. The skin and soft tissue infections were diagnosed according to clinical examination and positive cultures [3,23].

The infections were considered healthcare-associated if the patient had a history of hospital admission less than eight weeks before hospitalization. The rest of the infections were community-acquired.

The serum presepsin level was measured on admission using the chemiluminescent enzyme immunoassay method and routine laboratory tests (liver and renal biochemistry, complete blood count, INR, CRP). For presepsin determination, we used a PATHFAST^®^ presepsin analyzer (Mitsubishi Chemical Medience Corporation, Tokyo, Japan). The method’s detection limit was 20 pg/mL. Procalcitonin was evaluated using an immunoassay (Cobas 8000, Roche Diagnostics, Basel, Switzerland), and the limit of detection was 0.02 ng/mL.

### 2.3. Statistical Analysis

The data were analyzed with SPSS software v20.0 (SPSS Inc., Chicago, IL, USA) and MedCalc software (v11.4, Ostend, Belgium). Categorical variables were expressed as percentages and compared using the Chi-square test. All the continuous variables were non-normally distributed end were expressed as median and interquartile range. These variables were tested for normality using the Kolmogorov–Smirnov test. The continuous data were compared using the Mann–Whitney U test. The receiver operating characteristics (ROC) curve was analyzed, and the area under the curve (AUROC) was calculated to establish diagnostic accuracy. The factors associated with early diagnosis of infections in patients with ACLF were identified using univariate and multivariate logistic regression. Cox regression was used to evaluate if the serum biomarkers are risk factors for in-hospital mortality in patients with ACLF. Correlations were evaluated with Spearman’s correlation index. In this study, *p* < 0.05 was set as the significance level.

## 3. Results

### 3.1. Patients Characteristics

**Figure 1:**
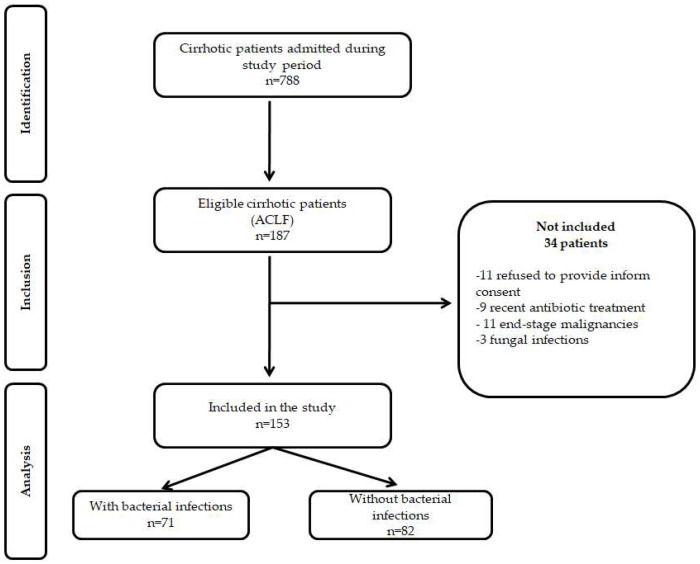
STROBE flow diagram of patients included and excluded from the study.

During the study period, 788 cirrhotic patients were admitted to our department, 203 were eligible for the study, and 153 patients fulfilled the inclusion criteria (Figure 1). There were no differences regarding gender, age, LC etiology, or severity between patients included in the study compared to those excluded.

The 153 patients included in this study had a median age of 60 years, and 65.4% of them were males. The main etiology of LC was alcoholic (87.6%), and 30 patients were diagnosed with acute alcoholic hepatitis (19.6%). Table 1 presents the characteristics of the participants. Most of the patients had comorbidities (72.5%). In the study group, 20.3% of the patients had variceal bleeding, and all of them were diagnosed with ascites. The majority of the patients (80.4%) had Child–Pugh C class liver cirrhosis, with a median MELD score of 26. More than half of the patients were diagnosed with ACLF grade 2 or 3 (52.9%). The median presepsin level in the study group was 2038 pg/mL, and a median procalcitonin level of 0.9 ng/mL.

### 3.2. Infectious Complications

Infections were diagnosed in 71 patients (46.4%), of whom 16 patients (22.5%) had more than two infections. Most patients had community-acquired infections (70.8%). There was no difference in the presence of alcoholic hepatitis in patients with or without infections (19.7% vs. 19.5%, *p* = 0.974).

The most frequent site of infection was UTI (28.2%), followed by SBP (26.8%), pneumonia (7.04%), and CDI (7.04%), and the Gram-negative bacteria were most frequently identified as etiologic agents (52.1%). Sepsis was diagnosed in 53 patients (74.6%) and was secondary to SBP, pneumonia, or UTI.

There was no significant difference between the two study groups in terms of age, gender, LC etiology, and comorbidities (Table 1). However, patients with infections had a more severe LC, as the MELD score (*p* = 0.002) and Child–Pugh score (*p* = 0.007) revealed. In the study group, 38.0% of the patients had chronic non-selective beta-blockers (NSBBs) treatment, and 14.1% had long-term proton pump inhibitors. There was no statistically significant difference between the patients with and without NSBBs treatment in terms of infection development (38.0% vs. 40.2%, *p* = 0.780). More than half of the patients had previous HE episodes (81 patients, 52.9%), and 61 patients (39.8%) received rifaximin as the HE secondary prophylaxis. There was no difference among the study groups in terms of previous rifaximin treatment (40.8% vs. 39.0%, *p* = 0.819).

### 3.3. Presepsin, PCT, and CRP Infectious Complications

The presepsin levels were higher in patients with infections compared to those without infections (6530 pg/mL vs. 1045 pg/mL, *p* < 0.001). Moreover, the presepsin levels increased with LC severity according to the Child–Pugh class (1265 pg/mL vs. 2290 pg/mL, *p* = 0.026), and there was a positive correlation between the presepsin levels and the MELD score (r = 0.336, *p* < 0.001).

The median CRP and PCT levels of the infections group were 5.65 mg/dL and 1.5 ng/mL respectively, and those without infections had a significantly lower median CRP of 3.03 mg/dL and median PCT of 0.7 ng/mL (*p* = 0.002 and *p* < 0.001, respectively) (Table 1). The median CRP levels in patients with class Child–Pugh B cirrhosis were not significantly different from median CRP levels in Child–Pugh C cirrhotic patients (3.65 mg/dL vs. 4.01 mg/dL, *p* = 0.854). PCT levels were higher in patients with Child–Pugh C liver cirrhosis compared to Child–Pugh B cirrhotic patients (0.7 ng/mL vs. 0.95 ng/mL, *p* = 0.027). There were no differences regarding presepsin and PCT levels between patients with Child C vs. Child–Pugh B liver cirrhosis independently of bacterial infections. The median level of presepsin in Child–Pugh B patients and infections was 4566 (5540) pg/mL compared to 5980 (5505) pg/mL in Child–Pugh C patients diagnosed with infections (*p* = 0.077). The median level of PCT in Child–Pugh B patients and infections was 1.1 (1.3) ng/mL compared to 1.5 (1.5) ng/mL in Child–Pugh C patients diagnosed with infections (*p* = 0.112).

**Figure 2:**
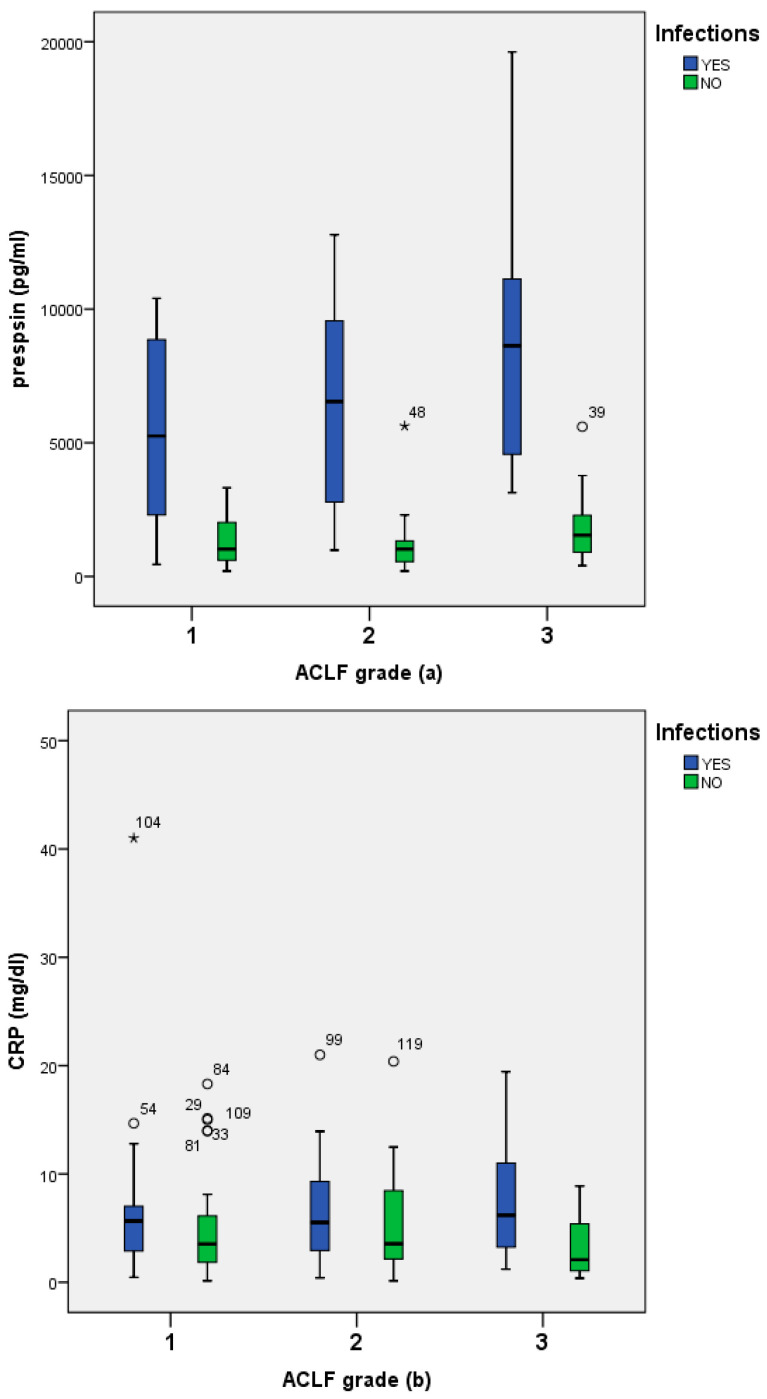

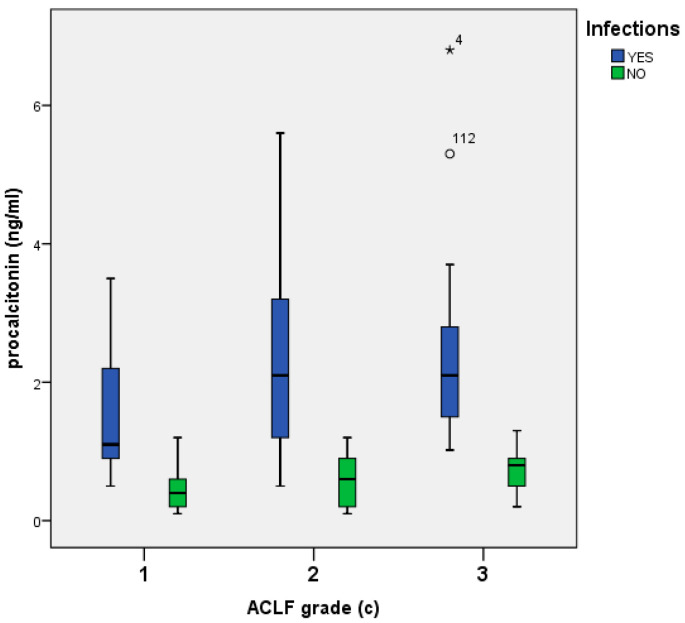
Presepsin (**a**), CRP (**b**), and procalcitonin (**c**) levels among patients with ACLF, with or without infections.

In patients with infections the median presepsin, CRP and PCT according to ACLF grade were as follows: 6530 (6280) pg/mL, 5.65 (6.99) mg/dL, and 1.5 (1.4) ng/mL in ACLF grade 1; 6440 (7082) pg/mL, 3.95 (5.24) mg/dL, and 1.1 (1.5) ng/mL in ACLF grade 2; and 7815 (5808) pg/mL, 6.2 (8.05) mg/dL, and 2.1 (1.4) ng/mL in ACLF grade 3, respectively (Figure 2).

### 3.4. Biomarkers Accuracy for Infection Diagnosis

**Figure 3:**
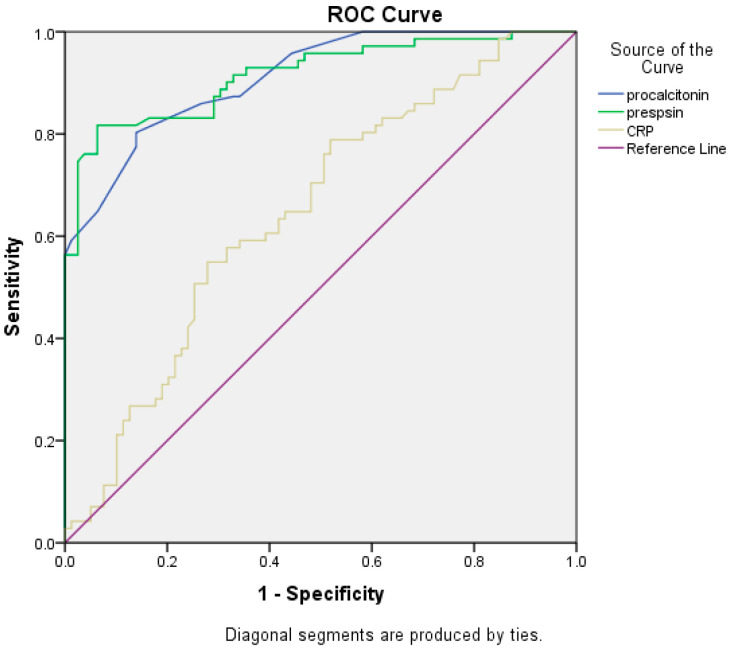
Comparisons of the three biomarkers’ ROC curves.

The ROC analysis was performed for three serum biomarkers: presepsin, CRP, and PCT. The results demonstrated that the best cut-off value for presepsin for infection detection was 2300 pg/mL with a sensitivity of 81.7%, and specificity of 92.7% (AUROC 0.911, CI 95%: 0.864–0.959, *p* < 0.001, Yourden index J = 0.743). For CRP the best cut-off value for infections detection was 5.3 mg/dL with a sensitivity of 54.9%, specificity of 69.6% (AUROC 0.648, CI 95%: 0.560–0.735, *p* < 0.002, Yourden index J = 0.458). Procalcitonin cut-off for infection diagnosis was 0.9 ng/mL, with a sensitivity of 80.3%, specificity of 86.6% (AUROC 0.909, CI 95%: 0.865–0.953, *p* < 0.001, Yourden index J = 0.668) (Figure 3). These results suggest that presepsin and PCT might have a diagnosis value for differentiating between ACLF patients with or without infections. The presepsin cut-off for infection diagnosis in patients with Child–Pugh B liver cirrhosis was 2290 pg/mL compared to 2304 pg/mL for patients with Child–Pugh C liver cirrhosis (*p* = 0.553). The PCT cut-off for infection diagnosis in patients with Child–Pugh B liver cirrhosis was 0.85 ng/mL compared to 0.95 ng/mL for patients with Child–Pugh C liver cirrhosis (*p* = 0.058).

Multivariate logistic regression analysis demonstrated that the presepsin level higher than 2300 pg/mL, PCT levels more than 0.9 ng/mL, and MELD score higher than 18 represent factors associated with the diagnosis of infections in patients with LC and ACLF (Table 2).

**Figure 4:**
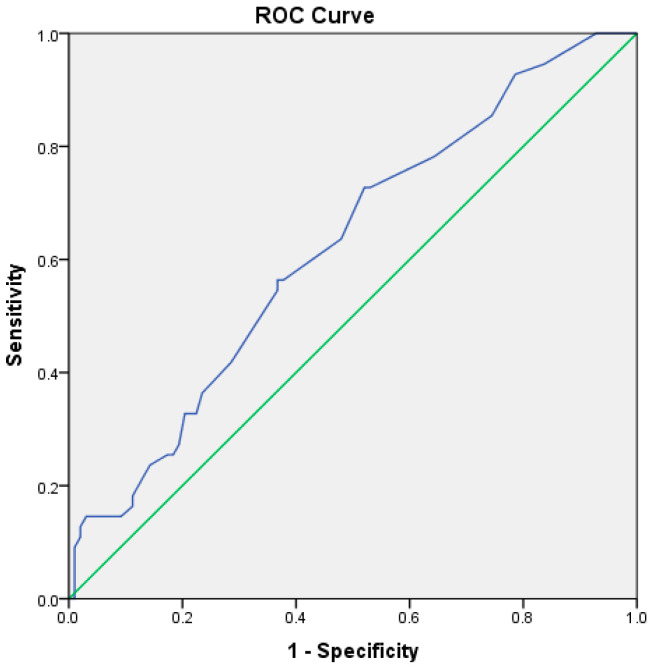
ROC curve for procalcitonin and in-hospital mortality (blue line—ROC curve, green line—reference line).

During hospitalization, 55 patients (35.9%) died. The mortality rate was not different in patients with ACLF and infections compared to those without infections (38.0% vs. 34.1%, *p* = 0.618). However, patients who died had higher baseline PCT levels than survivors (1.1 ng/mL vs. 0.8 ng/mL, *p* = 0.021), with no differences regarding presepsin or CRP levels (3225 pg/mL vs. 1994 pg/mL, *p* = 156, and 5.22 mg/dL vs. 3.65 mg/dL, *p* = 0.926, respectively). Most of the patients died during the first week after admission (37 patients, 67.3%). Procalcitonin cut-off for mortality during hospitalization was 0.9 ng/mL, with a sensitivity of 56.4%, specificity of 37.8% (AUROC 0.622, CI 95%: 0.531–0.712, *p* = 0.013) (Figure 4).

Presepsin, PCT, and CRP were not identified as risk factors for in-hospital mortality in our study (Table 3).

## 4. Discussion

Bacterial infections complicate the course of LC and determine an increased rate of decompensation, acute-on-chronic liver failure, and mortality [3]. The early diagnosis and prompt treatment of infections prevent LC from further decompensation and sepsis development and may decrease mortality rates [8,11,23]. Few studies compared the diagnostic role of different biomarkers to detect bacterial infections in patients with LC [9,11,17,20,24,25], and even fewer in patients admitted with ACLF [21,22].

ACLF is a very dynamic entity associated with a high mortality rate [1,2]. Bacterial infections, acute alcoholic hepatitis, and HBV flares are the main ACLF precipitating events, and early identification of them could decrease the mortality rates. Nowadays, there are three accepted ACLF definitions EASL-CLIF consortium, North American Consortium for the Study of End-Stage Liver Disease (NACSELD), and APASL, all of them including hepatic and/or extrahepatic organ failures in patients with acute decompensation of LC [1].

EASL-CLIF criteria include a combination of hepatic and extrahepatic organ failure variables precipitated by bacterial infections or alcoholic hepatitis. APASL-AARC criteria are based on predominantly hepatic failure variables precipitated by HBV flares or alcoholic hepatitis [1,26]. Considering these significant differences, the data regarding the utility of biomarkers in the diagnosis of infections in patients with ACLF could not be generalized to the European population. Therefore, we aimed to evaluate the role of presepsin, PCT, and CRP in the early diagnosis of bacterial infections in patients with ACLF, defined according to EASL-CLIF criteria.

The findings of the present study demonstrated that the serum presepsin level ≥2300 pg/mL had the most appropriate specificity and sensitivity to identify bacterial infections in patients admitted with ACLF. Moreover, the presepsin levels were directly correlated with LC severity assessed by the MELD score and the Child–Pugh class.

Prospective studies, including patients with compensated and decompensated LC, demonstrated that a presepsin level of >600 pg/mL was associated with an increased one-year liver-related mortality [10]. They also documented that cirrhotic patients have higher levels of presepsin even at the compensated stage and without infectious complications than presepsin levels previously reported in the general population. This fact could reflect spontaneous bacterial translocation associated with LC [13]. In our cohort, the main etiology of LC was chronic alcohol consumption. Chronic alcohol consumption is associated with increased intestinal permeability and a higher rate of endotoxin absorption, and this could explain the higher level of presepsin in our cohort.

In the general population, the presepsin level in adult subjects was 55–184 pg/mL, whereas in our study, the median presepsin level in patients without infections was 1045 pg/mL. This is in accordance with previous data that demonstrated a higher presepsin level in patients with LC without bacterial infections due to persistent intestinal bacterial translocation [10,27].

Papp et al. concluded that presepsin had better diagnostic accuracy in patients with LC and infections-associated organ failure. The association between CRP and presepsin increases the diagnostic accuracy of these biomarkers in terms of infection diagnosis in patients with LC [28]. They also demonstrated that the presepsin cut-off for infection diagnosis was 844 pg/mL, above the value previously reported for the general population (400–600 pg/mL) [14,29]. Furthermore, Novelli et al. showed that presepsin could discriminate bacterial infections from other types of infection with different cut-off values according to the type of infection [24].

Our results demonstrate significantly higher values of median presepsin compared to median presepsin levels reported in Asian cohorts for patients with ACLF and sepsis (508.5 pg/mL), confirming the fact that those data could not be translated in our cohorts [20].

In clinical practice, the diagnosis of infection should be set up in the context of characteristic symptoms and laboratory tests, including the presepsin level. Samples collections for culture should be obtained in each cirrhosis patient admitted with ACLF. However, the empirical antibiotic treatment could be considered if the presepsin level is higher than 2300 pg/mL. The goal is to prevent the evolution of sepsis and septic shock, considering that this diagnosis is challenging in patients with ACLF because they already have an altered mental state due to HE, leucopenia, arterial hypotension, or hypoxemia, secondary portal hypertension, and all the sepsis criteria include all these items.

In our study, CRP at a cut-off value of 5.3 mg/dL had a sensitivity of 54.9% and specificity of 69.6% in the diagnosis of infection. This cut-off is higher than those published before in patients with LC and bacterial infections but comparable with the results from the Asian cohorts, although lower specificity and sensibility could be explained by the excessive inflammation that characterizes ACLF and that promotes and sustains organ failure, not always associated with bacterial infections. Additionally, the differences between median CRP levels were not significant between ACLF grade 1 or 2 patients with or without bacterial infections.

According to our study, the median PCT level was significantly higher in patients with ACLF and infections compared to those without infections. Moreover, the overall performance of PCT in the diagnosis of infection in ACLF patients was superior to that of CRP and comparable with presepsin.

Zhang et al. [23] evaluated new biomarkers in the development of nosocomial infections in patients with ACLF. They demonstrated that there was no difference in PCT levels between patients with or without bacterial infections (0.42 ng/mL vs. 0.38 ng/mL, *p* = 0.260), although CRP levels were higher in patients with ACLF that developed bacterial infections (8.4 mg/dL vs. 14.4 mg/dL, *p* < 0.001) [21]. They also included IL-6, CRP, and serum globulin in a predictive model for bacterial infections in patients with ACLF.

In other Asian, Chen et al. [24] demonstrated that presepsin was significantly higher in patients with ACLF and sepsis compared to ACLF patients without bacterial infections (*p* < 0.001). They demonstrated a presepsin cut-off of 404.5 pg/mL for sepsis diagnosis, with an AUROC of 0.790, and a cut-off of 0.76 ng/mL for PCT, with an AUROC of 0.690 [22].

Multivariate regression demonstrated that only presepsin ≥2300 pg/mL, PCT ≥ 0.9 ng/mL, and MELD score ≥18 represent factors associated with infections in patients with ACLF. In the Asian cohort analyzed by Zhang et al., PCT was not recognized as a predictor factor for bacterial infections, and only serum globulin, CRP, and IL-6 were independent predictors for bacterial infections in patients with HBV-ACLF. These data could be explained by the differences in baseline cohorts’ characteristics, confirming the necessity of different management approaches according to ACLF precipitating factors.

The present study has some strengths and limitations. This is the first prospective cohort study examining the role of presepsin, reactive protein, and procalcitonin in the early diagnosis of infections in patients with ACLF defined according to EASL-CLIF criteria. The limitations of our research consist of the small number of patients and short follow-up period. Moreover, above 80% of the study population had the alcoholic etiology of LC, and our findings could not be transferred to patients with other LC etiologies. Considering that the data are not homogeneous, different cut-offs should be defined for diagnosing infections in patients with non-alcoholic LC and ACLF or other LC complications.

## 5. Conclusions

Presepsin and PCT may be adequate non-invasive tools for the early diagnosis of infections in patients with ACLF to decrease mortality rates during hospitalization. Assessment for infections in all admitted ACLF cirrhotic patients and early antibiotics administration are the keys to successful treatment and reversal of ACLF in patients with LC. In this regard, further prospective large cohort study should confirm the present findings.

## Figures and Tables

**Table 1:** Clinical and laboratory characteristics of the study groups.

Parameter	All Patients *n* = 153	With Bacterial Infections *n* = 71	Without Bacterial Infections *n* = 82	*p*
Age, years, median, (IQR)	60 (16)	60 (20)	59.5 (15)	0.418
Male sex, *n* (%)	100 (65.4)	45 (63.3)	55 (67.1)	0.632
Comorbidities, *n*, (%)	111 (72.5)	51 (71.8)	60 (73.1)	0.853
Cirrhosis alcoholic etiology, *n*, (%)	134 (87.6)	62 (87.3)	72 (87.8)	0.928
Acute alcoholic hepatitis, *n*, (%)	30 (19.6)	14 (19.7)	16 (19.5)	0.974
ACLF grade 1/2/3, *n*, (%)	72/39/42 (47.1/25.5/27.4)	33/17/21 (46.4/23.9/29.7)	39/22/21 (47.5/26.8/25.7)	0.839
Child–Pugh class B/C, *n*, (%)	30/123 (19.6/80.4)	11/60 (15.4/84.5)	19/63 (23.1/76.9)	0.233
Child–Pugh score median, (IQR)	12 (3)	12 (3)	11 (2)	**0.007**
MELD score, median, (IQR)	26 (13)	28 (11)	26 (17)	**0.002**
Variceal bleeding, *n*, (%)	31 (20.3)	11 (15.4)	20 (24.4)	0.172
Ascites grade 1/2/3, *n*, (%)	11/58/84 (7.2/37.9/54.9)	5/28/38 (7.04/39.4/53.56)	6/30/46 (7.3/36.6/56.)	0.936
CLIF ACLF score, median, (IQR)	55.2 ± 10.3	57.5 (13)	57 (14)	**0.049**
qSOFA score, median, IQR	1 (1)	2 (2)	1 (0)	**<0.001**
Total bilirubin, mg/dL, median, (IQR)	8.9 (9.6)	8.7 (13.1)	7.8 (5.08)	0.347
Lactate median, (IQR)	28 (18)	31.8 (15.6)	26.7 (26)	0.919
Creatinine mg/dL, median, (IQR)	2.23 (1.17)	2.23 (1.19)	2.09 (2.34)	0.723
INR, median, (IQR)	2.5 (0.88)	2.6 (0.76)	2.5 (0.23)	0.700
CRP, mg/dL, median (IQR)	4.07 (5.7)	5.65 (6.99)	3.36 (4.80)	**0.023**
Presepsin, pg/mL, median (IQR)	2038 (5314.5)	6530 (6385)	1045 (1715)	**<0.001**
Procalcitonin, ng/mL, median (IQR)	0.9 (1)	1.5 (1.5)	0.7 (0.75)	**<0.001**
NSBB *n*, (%)	60 (39.2)	27 (38.0)	33 (40.2)	0.780
PPI *n*, (%)	25 (16.3)	10 (14.1)	15 (18.3)	0.483
Rifaximin *n*, (%)	61 (39.9)	29 (40.8)	32 (39.02)	0.819
Hospitalization days, median (IQR)	15 (12)	15 (13)	15 (11)	0.544
In hospital mortality *n*, (%)	55 (35.9)	27 (38.0)	28 (34.1)	0.618

CRP, C-reactive protein; MELD, Model of End-Stage Liver Disease; NSBBs, non-selective beta-blockers; PPIs, proton pump inhibitors.

**Table 2:** Univariate and multivariate logistic regression analyses of factors associated with infections in patients with ACLF.

Parameter	Univariate Analysis			Multivariate Analysis		
	OR	CI 95%	*p*-Value	OR	CI 95%	*p*-Value
Male gender	0.94	0.749–1.193	0.758	0.75	0.278–2.050	0.581
Presepsin ≥ 2300 pg/mL	2.88	1.917–4.350	**<0.001**	3.65	1.394–9.588	**0.008**
CRP ≥ 5.3 mg/dL	1.64	1.129–2.405	**0.013**	2.07	0.789–5437	0.139
PCT ≥0.9 ng/mL	5.98	3.412–10.491	**<0.001**	8.79	6.168–25.736	**<0.001**
Acute alcoholic hepatitis	1.01	0.531–1.922	0.974	0.518	0.149–1.800	0.301
MELD ≥ 18	1.20	1.064–1.374	**0.008**	7.37	1.416–18.430	**0.018**
Child–Pugh class C	1.10	0.942–1.284	0.323	1.03	0.313–3.404	0.958
Previous NSBBs treatment	0.94	0.635–1.406	0.779	1.04	0.326–3.304	0.949
Previous rifaximin treatment	1.04	0.709–1.545	0.819	1.86	0.608–5.693	0.949
Previous PPIs treatment	0.77	0.369–1.605	0.481	1.27	0.417–3.928	0.667

CRP, C-reactive protein; PCT, procalcitonin; CI, confidence interval; OR, odds ratio; MELD, Model of End-Stage Liver Disease; NSBBs, non-selective beta-blockers; PPIs, proton pump inhibitors.

**Table 3:** Cox regression model for in-hospital mortality.

Parameter	B	Wald	RR	95% CI	*p*-Value
PCT ≥ 0.9 ng/mL	0.608	3.570	1.83	0.978–3.453	0.059
Presepsin ≥ 2300 pg/mL	0.116	0.137	1.12	0.607–2.07	0.711
CRP ≥ 5.3 mg/dL	0.269	0.885	1.30	0.747–2.289	0.347

CRP, C-reactive protein; PCT, procalcitonin; CI, confidence interval; RR, risk ratio.

## Data Availability

Data supporting reported results can be provided upon request in an electronic format.

## References

[1] Arroyo V., Moreau R., Jalan R. (2020). Acute-on-Chronic Liver Failure. N. Engl. J. Med..

[2] Moreau R., Jalan R., Gines P., Pavesi M., Angeli P., Cordoba J., Durand F., Gustot T., Saliba F., Domenicali M. (2013). Acute-on-Chronic Liver Failure Is a Distinct Syndrome That Develops in Patients with Acute Decompensation of Cirrhosis. Gastroenterology.

[3] Piano S., Singh V., Caraceni P., Maiwall R., Alessandria C., Fernandez J., Soares E.C., Kim D.J., Kim S.E., Marino M. (2019). Epidemiology and Effects of Bacterial Infections in Patients with Cirrhosis Worldwide. Gastroenterology.

[4] Kumar A., Roberts D., Wood K.E., Light B., Parrillo J.E., Sharma S., Suppes R., Feinstein D., Zanotti S., Taiberg L. (2006). Duration of hypotension before initiation of effective antimicrobial therapy is the critical determinant of survival in human septic shock. Crit. Care Med..

[5] Arvaniti V., D’Amico G., Fede G., Manousou P., Tsochatzis E., Pleguezuelo M., Burroughs A.K. (2010). Infections in Patients with Cirrhosis Increase Mortality Four-Fold and Should Be Used in Determining Prognosis. Gastroenterology.

[6] Piotrowski D., Boroń-Kaczmarska A. (2017). Bacterial infections and hepatic encephalopathy in liver cirrhosis–prophylaxis and treatment. Adv. Med. Sci..

[7] Villanueva C., Albillos A., Genescà J., Garcia-Pagan J.C., Brujats A., Calleja J.L., Aracil C., Bañares R., Morillas R.M., Poca M. (2021). Bacterial infections adversely influence the risk of decompensation and survival in compensated cirrhosis. J. Hepatol..

[8] Piano S., Bartoletti M., Tonon M., Baldassarre M., Chies G., Romano A., Viale P., Vettore E., Domenicali M., Stanco M. (2018). Assessment of Sepsis-3 criteria and quick SOFA in patients with cirrhosis and bacterial infections. Gut.

[9] Fischer P., Grigoras C., Bugariu A., Nicoara-Farcau O., Stefanescu H., Benea A., Hadade A., Margarit S., Sparchez Z., Tantau M. (2019). Are presepsin and resistin better markers for bacterial infection in patients with decompensated liver cirrhosis?. Dig. Liver Dis..

[10] Elefsiniotis I. (2018). Presepsin levels in cirrhotic patients with bacterial infections and/or portal hypertension-related bleeding, presenting with or without acute kidney injury. Ann. Gastroenterol..

[11] Ferrarese A., Frigo A.C., Mion M.M., Plebani M., Russo F.P., Germani G., Gambato M., Cillo U., Cattelan A., Burra P. (2021). Diagnostic and prognostic role of presepsin in patients with cirrhosis and bacterial infection. Clin. Chem. Lab. Med..

[12] Lin K.H., Wang F.L., Wu M.S., Jiang B.Y., Kao W.L., Chao H.Y., Wu J.-Y., Lee C.-C. (2014). Serum procalcitonin and C-reactive protein levels as markers of bacterial infection in patients with liver cirrhosis: A systematic review and meta-analysis. Diagn. Microbiol. Infect. Dis..

[13] Shozushima T., Takahashi G., Matsumoto N., Kojika M., Endo S., Okamura Y. (2011). Usefulness of presepsin (sCD14-ST) measurements as a marker for the diagnosis and severity of sepsis that satisfied diagnostic criteria of systemic inflammatory response syndrome. J. Infect. Chemother..

[14] Velissaris D., Zareifopoulos N., Karamouzos V., Karanikolas E., Pierrakos C., Koniari I., Karanikolas M. (2021). Presepsin as a Diagnostic and Prognostic Biomarker in Sepsis. Cureus.

[15] Yang H.S., Hur M., Yi A., Kim H., Lee S., Kim S.N. (2018). Prognostic value of presepsin in adult patients with sepsis: Systematic review and meta-analysis. PLoS ONE.

[16] Zhang X., Liu D., Liu Y.N., Wang R., Xie L.X. (2015). The accuracy of presepsin (sCD14-ST) for the diagnosis of sepsis in adults: A meta-analysis. Crit. Care.

[17] Bota D.P., Van Nuffelen M., Zakariah A.N., Vincent J.L. (2005). Serum levels of C-reactive protein and procalcitonin in critically ill patients with cirrhosis of the liver. J. Lab. Clin. Med..

[18] Kan W.C., Huang Y.T., Wu V.C., Shiao C.C. (2021). Predictive Ability of Procalcitonin for Acute Kidney Injury: A Narrative Review Focusing on the Interference of Infection. Int. J. Mol. Sci..

[19] Villarreal E., Vacacela K., Gordon M., Calabuig C., Alonso R., Ruiz J., Kot P., Babiloni D., Ramírez P. (2016). Usefulness of procalcitonin for diagnosing infection in critically ill patients with liver cirrhosis. Med. Intensiva Engl..

[20] Sato S., Sato S., Tsuzura H., Ikeda Y., Hayashida S., Takahashi S., Amano N., Murata A., Shimada Y., Iijima K. (2020). Elevated serum procalcitonin levels and their association with the prognosis of patients with liver cirrhosis. Eur. J. Gastroenterol. Hepatol..

[21] Zhang Z., Ma K., Yang Z., Cheng Q., Hu X., Liu M., Liu Y., Liu T., Zhang M., Luo X. (2021). Development and Validation of a Clinical Predictive Model for Bacterial Infection in Hepatitis B Virus Related Acute-On-Chronic Liver Failure. Infect Dis. Ther..

[22] Chen J., Ze-Bing H., Li H., Zheng X., Chen J.J., Wang X.B., Qian Z., Liu X., Fan X., Hu X. (2021). Early Diagnostic Biomarkers of Sepsis for Patients with Acute-on-Chronic Liver Failure: A Multicenter Study. Infect. Dis. Ther..

[23] European Association for the Study of the Liver (2018). EASL Clinical Practice Guidelines for the management of patients with decompensated cirrhosis. J. Hepatol..

[24] Novelli S., Morabito V., Ruberto F., Bini F., Marinozzi F., Pugliese F., Berloco P., Pretagostini R. (2020). Diagnostic Value of Presepsin for Bacterial Infection in Cirrhosis: A Pilot Study. Transplant. Proc..

[25] Khedher S., Fouthaili N., Maoui A., Lahiani S., Salem M., Bouzid K. (2018). The Diagnostic and Prognostic Values of C-Reactive Protein and Procalcitonin during Bacterial Infections in Decompensated Cirrhosis. Gastroenterol. Res. Pract..

[26] Zaccherini G., Weiss E., Moreau R. (2021). Acute-on-chronic liver failure: Definitions, pathophysiology and principles of treatment. JHEP Rep..

[27] Fukui H. (2021). Leaky Gut and Gut-Liver Axis in Liver Cirrhosis: Clinical Studies Update. Gut Liver.

[28] Papp M., Tornai T., Vitalis Z., Tornai I., Tornai D., Dinya T., Sumegi A., Antal-Szalmas P. (2016). Presepsin teardown—Pitfalls of biomarkers in the diagnosis and prognosis of bacterial infection in cirrhosis. World J. Gastroenterol..

[29] Ulla M., Pizzolato E., Lucchiari M., Loiacono M., Soardo F., Forno D., Morello F., Lupia E., Moiraghi C., Mengozzi G. (2013). Diagnostic and prognostic value of presepsin in the management of sepsis in the emergency department: A multicenter prospective study. Crit. Care.

